# Evaluation of β-thalassemias in the premarital hemoglobinopathy screening program: A retrospective study

**DOI:** 10.12669/pjms.41.2.10024

**Published:** 2025-02

**Authors:** Olgun Goktas

**Affiliations:** 1 Dr. Olgun Goktas Associate Professor, Uludag University Family Health Center, 16059, Gorukle Campus-Nilufer, Bursa, Turkey

**Keywords:** β-thalassemia, Hemoglobinopathy, Premarital screening, Prenatal testing, Primary care

## Abstract

**Objective:**

To retrospectively evaluate β-thalassemias in the premarital hemoglobinopathy screening program in primary care.

**Methods:**

The retrospective study was carried out in Bursa Uludag University Family Health Center in Turkey between 1-30 September 2023. In the study, the data of individuals who applied to the Family Health Center for premarital health examination within the four years between January 1, 2019, and December 31, 2022, were taken from the database and evaluated retrospectively. Family history of hemoglobinopathy, sociodemographic findings, existing diseases, allergies, cancer, and genetic disease conditions were obtained. Complete blood count, and high-performance liquid chromatography (HPLC) results were examined. *P-values* below 0.05 were considered statistically significant. Analyzes were made in the SPSS 25.0 program.

**Results:**

A total of 327 people, 171 men (52.3%) and 156 (47.7%) women, participated in the study. It was determined that the age of the individuals was 30.17±6.16. The average Mentzer index levels were found to be 12.95±4.56. Places of birth are Mediterranean with 8.3% and other regions with 91.7%. β-thalassemia type detected in family medicine was suspected with a rate of 1.5% and carrier with a rate of 0.6%. The rate of patients referred to a hematologist was found to be 2.1%. The rate of patients with a definitive diagnosis was determined as 1.8%. It was determined that β-thalassemia definitive diagnosis rates were higher in groups whose place of birth was the Mediterranean region, with a family history of thalassemia, with a diagnosis of cancer, and with a genetic, allergic, and chronic disease diagnosis (p=0.01).

**Conclusion:**

Although it is not located in the Mediterranean region, the high prevalence of β-thalassemia in our population and its relationship with diseases are important. We emphasize the importance of a premarital screening program for the diagnosis of β-thalassemia due to its increasing frequency and complications in the globalizing world.

## INTRODUCTION

Hemoglobinopathies, the most common genetic blood diseases, are an important health problem all over the world. Although it is more common, especially in the Mediterranean and surrounding regions, its incidence is increasing in other regions due to current migration events. They can cause growth and developmental delay, hepatosplenomegaly, jaundice, affect other organs, and lead to mild to severe anemia that lasts a lifetime. Since it is a genetically inherited disorder, hemoglobinopathies must be detected in advance with a premarital screening program. In family medicine practice, it is important to screen prospective spouses for marriage, diagnose these diseases in advance, and take the necessary precautions.

Thalassemias, included in hemoglobinopathies, is a heterogeneous group of genetic disorders resulting from decreased synthesis of alpha or beta chains of hemoglobin (Hb). β-thalassemia is caused by point mutations in the beta-globin gene. It causes anemia that begins in early childhood and lasts throughout life. Thalassemia is a hereditary disease and at least one of the parents must be a carrier for this disease. It may present clinically with jaundice, growth failure, hepatosplenomegaly, endocrine abnormalities, and severe anemia requiring lifelong blood transfusions.^1^-^4^

Taking into account risky areas, the Premarital Hemoglobinopathy Screening Program was put into practice in family medicine by the Ministry of Health in Turkey at the end of 2018. The frequency of β-thalassemia carriers in Turkey is 2.1% and it is reported that there are approximately 1300000 carriers and around 4500 patients. Depending on the carrier frequency, 300-400 sick children are expected to be born annually. The damage it will cause to the family and society is important, not only in terms of health but also in economic and social terms.^5^

Although hemoglobinopathies are serious chronic diseases with few treatment options, timely detection of carriers makes it possible to protect those at risk from the disease and its consequences. Pre-pregnancy and prenatal diagnosis and necessary interventions enable conscious reproduction. The prevalence of hemoglobinopathy, which increased due to immigrants in Northern Europe, was also shown to increase in immigrant neighborhoods in a study conducted in The Hague city of the Netherlands. Despite the high carrier prevalence, it is stated that new research is needed to diagnose sickle cell anemia and thalassemia.^6^-^8^

In a six years retrospective study conducted on approximately 100000 people in Saudi Arabia, where the prevalence of Thalassemia is high, the results of sickle cell anemia and Thalassemia carriage were evaluated with the blood samples taken from the spouses during the Hemoglobinopathy screening performed before pregnancy. Genetic counseling was given to the spouses. At the end of six years, while the prevalence of sickle cell anemia remained constant, the number of hemoglobinopathy diseases decreased five fold thanks to voluntary pregnancy cancellations. It is stated that this decrease will reduce the number of genetic diseases in the coming years.^9^

Despite the latest developments in thalassemia treatment, deficiencies in service provision and inadequacy of systems due to the increase in the global population increase the need for a total approach.^10^ In a study conducted in Pakistan reporting an increase in common molecular anomalies in β-thalassemia disease, a premarital screening program is strongly recommended.^11^ In a study conducted in Pakistan, it is emphasized that it is important to determine hemoglobinopathies at the national level and it is recommended to confirm the diagnoses with premarital screening programs and high-performance liquid chromatography (HPLC) method to be applied in the hospital.^12^ The importance of national premarital screening programs is emphasized due to the increasing prevalence of β-thalassemia in the USA^13^, and the importance of primary care in the decision-making phase in the Netherlands.^14^

Hemoglobinopathies, β-thalassemia, and genetic diseases will increase soon, not only in the Mediterranean and surrounding regions but also on a global basis, if precautions are not taken due to increasing internal and external migration. The Premarital Hemoglobinopathy Screening Program implemented in family medicine should be continued. In addition, it is important that the family physician adequately informs the spouses during the premarital evaluation phase and raises awareness about the consequences. In this study, we aimed to evaluate the β-thalassemia status and the relationships between possible factors in the Premarital Hemoglobinopathy Screening Program recorded within the specified period in individuals registered to the family health center.

## METHODS

This retrospective study was carried out at Bursa Uludağ University Family Health Center between 1-30 September 2023. In the study, the data of individuals who applied to the Family Health Center for premarital health examination within the four years between January 1, 2019, and December 31, 2022, were taken from the database (recorded in the Family Medicine Information Registration System) and evaluated retrospectively. Individuals registered to the family health center participated in the study. Those who migrated out of the region and those who were not registered were not included in the study.

Patient records, which are secondary data sources, were used as a data collection method. Individual data such as age, gender, place of birth, family history of hemoglobinopathy, allergy, cancer, genetics, chronic illnesses, and presence of conditions associated with high HbA2 during the research period were obtained from the family medicine information registration system anonymously. As laboratory blood test results, complete blood count, serum iron and ferritin levels, and high-performance liquid chromatography (HPLC) test results were obtained. The Mentzer index (RBC: Red blood cell / MCV: Mean corpuscular volume) of the individuals was calculated. The current findings of those who were found to be thalassemia carriers or diseases were analyzed.

### Ethical approval:

The study was performed after approval from the Clinical Research Ethics Committee of Bursa Uludağ University, Faculty of Medicine (Ref. Dated 2023-08-01/ decision no: 2023-16/13) following the Declaration of Helsinki.

### Statistical analysis:

In the study, numerical data obtained from the cases were coded and transferred to the computer program. Descriptive statistics are given with mean, deviation, frequency, and percentage values. The Chi-square test and Mann-Whitney U test were used to examine the demographic and clinical characteristics of the patients according to their definitive diagnosis status. ROC (Receiver-Operating Characteristic) analysis was performed and ROC curves were drawn to examine the diagnostic power of preliminary diagnosis and definitive diagnosis levels regarding thalassemia. AUROC (Area under the Receiver Operating Characteristic) values were calculated to compare ROC areas. SPSS (Statistical Package for Social Science, Chicago, II, USA) 25.0 Windows package program was used for statistical evaluation.

## RESULTS

A total of 327 people, 171 men (52.3%) and 156 (47.7%) women, participated in the study. Places of birth are Mediterranean with 8.3% and other regions with 91.7%. Among the participants, there was a history of thalassemia with a rate of 1.5%, a genetic disease with a rate of 0.9%, a diagnosis of cancer with a rate of 0.6%, a diagnosis of allergic disease with a rate of 3.4%, a diagnosis of chronic disease with a rate of 8.6%, diagnosis of non-β-thalassemia blood disease with a rate of 0.3%, and the presence of conditions associated with high HbA2, with a rate of 3.1% was found (Table-I).

**Table-I:** General characteristics and comorbidities of participants

		n	%	
Gender	Male	171	52.3%
	Female	156	47.7%
Place of birth	Mediterranean region and surroundings	27	8.3%
	Other	300	91.7%
Number of family members with a history of thalassemia	Yes	5	1.5%
History of thalassemia in the prospective spouse	Yes	0	0.0%
Genetic disease diagnosis	Yes	3	0.9%
Cancer disease diagnosis	Yes	2	0.6%
Allergic disease diagnosis	Yes	11	3.4%
Chronic disease diagnosis	Yes	28	8.6%
Diagnosis of non-β-thalassemia blood disease	Yes	1	0.3%
Presence of conditions associated with high HbA2	Yes	10	3.1%

It was determined that the age of the individuals was 30.17±6.16. The average Mentzer index levels were found to be 12.95±4.56. (Table-II) In the HPLC analysis, it was determined that there was high HbA2 at 2.8%, low HbA at 0.9%, presence of HbF at 1.5%, presence of HbS at 0.6%, presence of HbC at 0% and presence of Hb0 at 0.9%. According to laboratory full blood results, Erythrocyte (RBC) was high or normal by 4.3%, MCV was low by 7%, MCH was low by 8%, RDW was slightly high or normal by 3.7%, Hb was 7% and Htc was low by 5.8%. When other laboratory blood results were examined, serum iron levels were 4% low and Ferritin levels were 1.5% low. (Table-III) β-thalassemia type detected in family medicine was suspected with a rate of 1.5% and carrier with a rate of 0.6%. The rate of patients referred to a hematologist was found to be 2.1%. The rate of patients with a definitive diagnosis was determined as 1.8% (Table-IV).

**Table-II:** Participants’ age and Mentzer indices results.

	X±SD (mean±SD)
Age	30.17±6.16
Mentzer index (MCV/RBC)	12.95±4.56

MCV: Mean corpuscular volume, RBC: Red blood cell count.

**Table-III:** HPLC. CBC. and other blood results in the laboratory.

			n	%
Height HbA2		Yes	9	2.8%
Low HbA		Yes	3	0.9%
Presence of HbF		Yes	5	1.5%
Presence of HbS		Yes	2	0.6%
Presence of HbC		Yes	0	0.0%
Presence of Hb0		Yes	3	0.9%
Erythrocyte (RBC) high or normal		Yes	14	4.3%
Low MCV		Yes	23	7.0%
Low MCH		Yes	26	8.0%
RDW slightly high or normal		Yes	12	3.7%
Low Hb		Yes	23	7.0%
Low Htc		Yes	19	5.8%
Laboratory other blood result	No		309	94.5%
	Low serum iron		13	4.0%
	Serum total iron binding capacity		0	0.0%
	Low ferritin		5	1.5%

HPLC: High-performance liquid chromatography. CBC: Complete blood count. RDW: Red cell distribution width.

**Table-IV:** Clinical evaluation and results, thalassemia screening, Mentzer index, hospital referral and diagnostic results.

		n	%
***Mentzer index (%);*** ≤13 = thalassemia >13 = iron deficiency anemia
	≤13	5	83.3%
	>13	1	16.7%
Type of β-thalassemia diagnosed in family medicine	Normal	320	97.9%
	Suspected	5	1.5%
	Carrier	2	0.6%
Referral to hematologist and geneticist	Yes	7	2.1%
Type of β-thalassemia with Definitive Diagnosis	Normal	321	98.2%
	Carrier	6	1.8%

It was determined that gender did not show a significant difference in terms of definitive diagnosis β-thalassemia levels (p = 0.26). It was also determined that β-thalassemia definitive diagnosis rates were higher in groups whose place of birth was the Mediterranean region, with a family history of thalassemia, with a diagnosis of cancer, and with a genetic, allergic, and chronic disease diagnosis (p = 0.01). It was found that β-thalassemia definitive diagnosis rates were higher in the group diagnosed with other blood diseases and in the group with conditions associated with high HbA2 (p = 0.01). β-thalassemia definitive diagnosis rates were found to be higher in the group with low HbA, presence of HbS, and low Htc (p = 0.01). On the other hand, β-thalassemia definitive diagnosis rates were found to be no different from other laboratory blood results according to serum iron and ferritin levels (p = 0.52). The definitive diagnosis results of β-thalassemia types (Suspected and carrier patients) detected in family medicine in secondary or tertiary health institutions highly supported the diagnosis of β-thalassemia (Table-V).

**Table-V:** Examination of general characteristics of patients according to Definitive Diagnosis.

		Definitive Diagnosis				p

		Normal		Carrier		

		n	%	n	%	
Gender	Male	168	52.3%	3	50.0%	0.26
	Female	153	47.7%	3	50.0%	
Place of birth	Mediterranean	25	7.8%	2	33.3%	0.01*
	Other	296	92.2%	4	66.7%	
Number of family members with a history of Thalassemia	No	321	100.0%	1	16.7%	0.01*
	Yes	0	0.0%	5	83.3%	
Genetic disease diagnosis	No	319	99.4%	5	83.3%	0.01*
	Yes	2	0.6%	1	16.7%	
Cancer disease diagnosis	No	321	100.0%	4	66.7%	0.01*
	Yes	0	0.0%	2	33.3%	
Allergic disease diagnosis	No	312	97.2%	4	66.7%	0.01*
	Yes	9	2.8%	2	33.3%	
Chronic disease diagnosis	No	296	92.2%	3	50.0%	0.01*
	Yes	25	7.8%	3	50.0%	
Diagnosis of non-β-thalassemia blood disease	No	320	99.7%	6	100.0%	0.01*
	Yes	1	0.3%	0	0.0%	
Presence of conditions associated with high HbA2	No	313	97.5%	4	66.7%	0.01*
	Yes	8	2.5%	2	33.3%	
Low HbA	No	319	99.4%	5	83.3%	0.01*
	Yes	2	0.6%	1	16.7%	
Presence of HbF	No	320	99.7%	5	83.3%	0.01*
	Yes	1	0.3%	1	16.7%	
Low Htc	No	306	95.3%	2	33.3%	0.01*
	Yes	15	4.7%	4	66.7%	
Laboratory other blood result	No	306	95.3%	3	50.0%	0.52
	Low serum iron	11	3.4%	2	33.3%	
	Low ferritin	4	1.2%	1	16.7%	
Mentzer index (%); ≤13 = thalassemia >13 = iron deficiency anemia
	≤13	0	0.0%	5	100.0%	-
	>13	1	100.0%	0	0.0%	
Type of β-thalassemia diagnosed in family medicine	Normal	320	99.7%	0	0.0%	0.01*
	Suspected	1	0.3%	4	66.7%	
	Carrier	0	0.0%	2	33.3%	
Referral to hematologist and geneticist	No	320	99.7%	0	0.0%	-
	Yes	1	0.3%	6	100.0%	

**Chi-Square analysis,

* significant difference at 0.05 level.

We also found that β-thalassemia carrier patients detected in family medicine were older than suspected patients (p = 0.01). The study also showed that the Mentzer index levels of the same patients were lower than the suspicious patients (p = 0.03). After a referral from the family physician, it was noted that patients with a definitive diagnosis of the carrier in secondary or tertiary health institutions were older than patients with a suspected diagnosis (p = 0.03). We also found that the Mentzer index of the same patients was lower than the suspicious patients (p = 0.01) (Table-VI).

**Table-VI:** β-thalassemia type determined according to measurements and those with a definitive diagnosisas a result of referral.

	Type of β-thalassemia diagnosed in family medicine			Definitive Diagnosis		

	Suspected	Carrier	p	Normal	Carrier	p

	X±SD (mean±SD)	X±SD (mean±SD)		X±SD (mean±SD)	X±SD (mean±SD)	
Age	29.8±6.83	43.5±13.44	0.01*	30.08±6.05	35.5±10.01	0.03*
Mentzer index (MCV/RBC) result	13.55±5.38	11.47±1.68	0.03*	22.98±1.01	11.28±1.24	0.01*

**Mann Whitney U test analysis,

* significant difference at 0.05 level.

**Fig.1:**
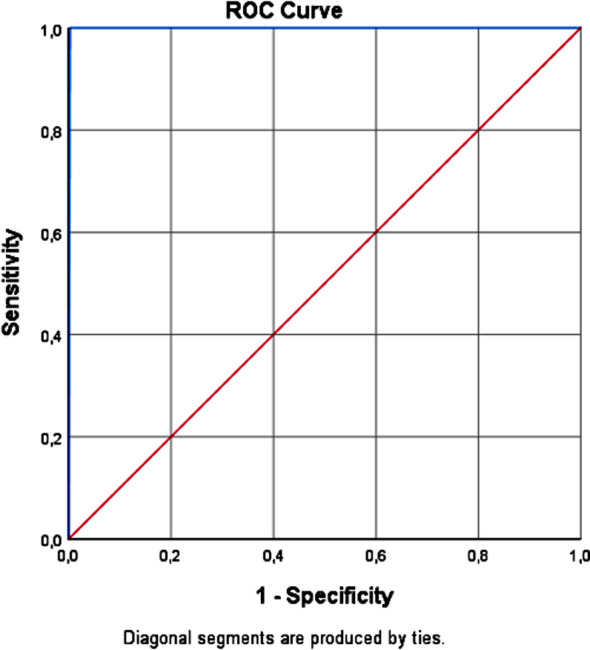
Comparison of preliminary diagnosis and definitive diagnosis efficiency ROC curve.

The study showed that the agreement between the preliminary diagnosis and the subsequent blood test diagnosis was quite high (ROC = 0.99) (Sensitivity = 0.99, Specificity = 0.98). The preliminary diagnoses in family medicine were largely correct (Fig.1).

### Sample, statistical power values, and strengths of the study:

**Fig.2:**
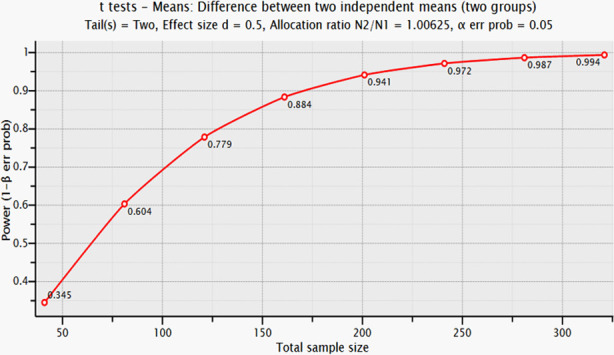
Sample sizes versus statistical power values.

To the best of our knowledge, no previous study has examined our primary outcomes. Therefore, an a priori power analysis was performed to determine the required sample size for our research. Since the effect sizes were unknown, we followed Cohen’s guidelines (Cohen, 1988)^15^ and considered a medium effect size (*d* = 0.5). One of the primary goals of our study is to compare the Mentzer index across definitive diagnoses; therefore, we based our power analysis on an independent samples t-test design. Calculations were carried out using G*Power statistical software with an alpha level of 0.05 and a desired power of 80%, which is the commonly accepted minimum threshold for adequate power. The results of the power analysis indicated that a total sample size of *n* = 128 participants (64 per group) would be required to detect a medium effect size with 80% power. However, our study included *n* = 327 participants, which exceeds the required minimum and provides an estimated power of 99.4%. The output of our power analysis is shown in Fig.2.

## DISCUSSION

Hemoglobinopathies are clinically important because they cause severe jaundice, growth and developmental delays, and lifelong severe anemia. Lifelong physician monitoring and, when necessary, blood transfusion and complications are important from a socioeconomic perspective as well as health perspective. There is no study in the literature examining the primary results of our study, and this creates a gap.

In this study, the rate of patients diagnosed with β-thalassemia during premarital hemoglobinopathy screening at the family health center was found to be 1.8%. The frequency of β-thalassemia carriers throughout the country is 2.1%. Although the family health center is not located in the Mediterranean and the surrounding region, the rate of patients is high. These rates will increase due to internal and external migration in the globalizing world. Β-thalassemia and other hemoglobinopathies need to be diagnosed with a premarital screening program because they will affect the whole life and have comorbidities and complications. The holistic approach of family medicine in primary prevention and the screening program it will implement are important in the diagnosis of β-thalassemia and similar hemoglobinopathies.

Individuals and society need to be aware of the problems that genetic disorders such as Β-thalassemia may cause after marriage. A study among parents of children with thalassemia reports better knowledge of male gender, higher education, and income level.^16^ The stress that thalassemia creates on the patient and the caregiver due to long-term service is important. It is stated that early diagnosis and precautions with premarital screening are economical.^17^ The birthplace of 8.3% of the population in our study was the Mediterranean region and its immediate surroundings. The birthplace of the other 91.7 of our population was placed outside the Mediterranean region. In our study, the diagnosis of Β-thalassemia was found more frequently in those born in the Mediterranean region and in those with a family history of thalassemia. In a study, it is stated that although β-thalassemia is more common in the Mediterranean, Middle-East, and Southeast Asian countries, its prevalence has increased over time, especially in Northern European and North American countries, due to migration.^18^

In a study conducted at a premarital screening center, it was stated that undiagnosed hemoglobinopathies and other anemias were at a significant level. In the same study, it is emphasized that iron deficiency anemia should be distinguished in the same screening program as well as identifying different types of hemoglobinopathies.^19^ Similar studies also state that the Mentzer index^20^ is useful in distinguishing β-thalassemia from iron deficiency anemia.^21^,^22^ In our study, the average Mentzer index levels were found to be 12.95. Only one suspicious patient had a Mentzer index >13 and was considered a normal individual as a result of referral to a hematologist. In our study, β-thalassemia carriers detected in family medicine had a lower Menzter index than suspects.

In our study, conditions such as vitamin B12 deficiency, folic acid deficiency, hyperthyroidism, antiretroviral treatment, calcium deficiency, vitamin C deficiency, and lead nephropathy, which may be related to high HbA2 levels that support Β-thalassemia, were also recorded. In the presence of these conditions, β-thalassemia definitive diagnosis rates were high. Similarly, β-thalassemia diagnosis rates were higher in those with low HbA and Htc. On the other hand, no significant difference was found in the definitive β-thalassemia diagnosis rates according to serum iron and ferritin levels.

In a premarital screening program conducted in Saudi Arabia, serum iron and ferritin tests were performed in individuals with low hemoglobin (Hb) to differentiate between iron deficiency anemia (IDA) and beta-thalassemia trait (β-TT). In addition to MCV, RBC, and red cell distribution width (RDW) values, the red cell distribution width index (RDWI) was calculated. At the end of the study, it is stated that RDWI is a reliable and useful index for the distinction between IDA and βTT.^23^ In another study, β-thalassemia carriers were detected by applying high-performance liquid chromatography (HPLC) to a woman without iron deficiency who applied to a tertiary obstetrics clinic. Subsequently testing the spouses of women who are carriers increases the success of the screening program.^23^ Interestingly, β-thalassemia disease can sometimes be detected suddenly. A patient who applied to the emergency department with abdominal pain for one week had splenomegaly detected by ultrasound. The diagnosis was confirmed by serum electrophoresis. The study emphasizes that, although rare, the presence of β-thalassemia should not be ignored in complications associated with this type of anemia.^24^

In our study, it was determined that gender did not differ significantly in those with a definitive diagnosis of β-thalassemia. On the other hand, β-thalassemia diagnosis was more common in those with cancer, genetic, allergic, and chronic diseases. In one study, it was reported that as survival time increases in β-thalassemia patients, there is an increase in unknown complications, especially hepatocellular carcinoma.^24^ In a study, it was determined that endocrine complications and thyroid diseases increased in patients with β-thalassemia.^25^

According to the World Health Organization, hemoglobin disorders occur in approximately 7% of pregnant women.^26^ The prevalence of β-thalassemia in prenatal women in the Uttakrand region of India is reported to be 2.6%. In our study, β-thalassemia type detected in family medicine was suspicious with a rate of 1.5%, and carrier with a rate of 0.6%. The rate of patients referred to a hematologist for definitive diagnosis was found to be 2.1%. The rate of β-thalassemia patients with a definitive diagnosis was determined as 1.8%.

In our study, it was determined that the agreement between the preliminary diagnoses in family medicine and the final diagnoses made by the hematologist after referral was quite high (ROC = 0.99). Sensitivity and specificity levels are also quite high (Sensivity = 0.99, Specificity = 0.98). It shows that the preliminary diagnoses determined in family medicine are largely correct. In our study, important results were obtained regarding the frequency of β-thalassemia and other hemoglobinopathies in our society and their relationship with the disease and sociodemographic characteristics. New studies are needed on the status of β-thalassemia and other hemoglobinopathies by region and country due to increasing internal and external migration as a result of globalization.

### Limitations:

Since our study is retrospective, the screening results were performed in different clinics while outside the region for various reasons and the fact that the data of individuals with thalassemia diagnosed were not included in our records may have created a limitation. The data of married patients with thalassemia disease and single thalassemia patients who did not attend marriage screening could not be evaluated. Data regarding other hemoglobinopathies and blood diseases other than beta thalassemia were not evaluated in the study.

## CONCLUSION

Although it is not located in the Mediterranean region, the high prevalence of β-thalassemia in our population and its relationship with diseases are important. We emphasize the importance of a premarital screening program for the diagnosis of β-thalassemia due to its increasing frequency and complications in the globalizing world.
